# Liver but not adipose tissue is responsive to the pattern of enteral feeding

**DOI:** 10.1002/phy2.250

**Published:** 2014-02-25

**Authors:** Yolanda F. Otero, Tammy M. Lundblad, Eric A. Ford, Lawrence M. House, Owen P. McGuinness

**Affiliations:** 1 Molecular Physiology and Biophysics Department, School of Medicine, Vanderbilt University, Nashville, Tennessee; 2 College of Medicine, University of Tennessee Health Science Center, Memphis, Tennessee

**Keywords:** Enteral delivery, feeding pattern, lipogenesis, nutritional support

## Abstract

Nutritional support is an important aspect of medical care, providing calories to patients with compromised nutrient intake. Metabolism has a diurnal pattern, responding to the light cycle and food intake, which in turn can drive changes in liver and adipose tissue metabolism. In this study, we assessed the response of liver and white adipose tissue (WAT) to different feeding patterns under nutritional support (total enteral nutrition or TEN). Mice received continuous isocaloric TEN for 10 days or equal calories of chow once a day (Ch). TEN was given either at a constant (CN, same infusion rate during 24 h) or variable rate (VN, 80% of calories fed at night, 20% at day). Hepatic lipogenesis and carbohydrate‐responsive element‐binding protein (ChREBP) expression increased in parallel with the diurnal feeding pattern. Relative to Ch, both patterns of enteral feeding increased adiposity. This increase was not associated with enhanced lipogenic gene expression in WAT; moreover, lipogenesis was unaffected by the feeding pattern. Surprisingly, leptin and adiponectin expression increased. Moreover, nutritional support markedly increased hepatic and adipose FGF21 expression in CN and VN, despite being considered a fasting hormone. In summary, liver but not WAT, respond to the pattern of feeding. While hepatic lipid metabolism adapts to the pattern of nutrient availability, WAT does not. Moreover, sustained delivery of nutrients in an isocaloric diet can cause adiposity without the proinflammatory state observed in hypercaloric feeding. Thus, the liver but not adipose tissue is responsive to the pattern of feeding behavior.

## Abstract (precis)

In the enteral feeding setting, hepatic lipogenesis responds to feeding pattern while the adipose tissue remains insensitive.

## Introduction

Clinicians rely on nutritional support to meet the metabolic demands of patients incapacitated by illness or injury. Consequently, patients often experience hyperglycemia, which is associated with increased morbidity and mortality in both diabetic and nondiabetic patients (McMahon et al. 2013). The incidence of hyperglycemia results from an interplay between an underlying stress and the high carbohydrate content of nutritional support. Thus, patients require nutritional support risk deleterious and potentially life threatening consequences. Importantly, in 2003, $3.1 billion was spent on nutritional support just in the United States of America, and the number has since doubled (Correia and Waitzberg 2003; Pfuntner et al. 2006). Given the expense and the high use of this medical practice, it is critical that we learn how to administer nutritional support to maximize patient outcomes.

The emergence of secondary pathologies during nutritional support results, in part, from a disruption in the normal diurnal feeding pattern. The resultant changes in body weight by disrupting the feeding pattern (Sukumaran et al. 2010) are accompanied by distinct adaptations in liver and adipose tissue metabolism. Specifically, previous work from our group described that the liver quickly adapts to a continuously infused carbohydrate diet by becoming an efficient consumer of glucose (Chen et al. 2000), independent of the route of delivery (Chen et al. 2005b). Indeed, manipulating the pattern of nutrient delivery alters liver expression of genes involved in fat and glucose metabolism. Studies carried out in mice under time‐restricted feeding demonstrated an interaction between feeding pattern and circadian rhythm that controls the hepatic gene expression of *Creb, Srebp1/2*,* Pparα* and *Ucp‐2* among others (Vollmers et al. 2009; Hatori et al. 2012; Jang et al. 2012). On the other hand, there is no information about the response of white adipose tissue to feeding pattern and nutritional support in an isocaloric diet. When rats were overfed with a high fat diet via enteral nutrition (Shankar et al. 2010), adiposity increased as well as adipokine secretion (PAI‐1). Also, there were changes in adipogenic gene expression (i.e. increased *Thedc1*,* Elovl5,* and *Gpam*) that might be mediated by carbohydrate‐responsive element‐binding protein (ChREBP). In another study in mice fed with high fat diet, disrupting the feeding pattern decreased fatty acid synthase (FAS) protein expression, activated AMP‐activated protein kinase (AMPK), and inhibited the pentose phosphate pathway (Stucchi et al. 2012).

The differential metabolic response of the liver and adipose tissue to nutritional status is also pronounced in the transition from fed to fasted state. Both serve as important sites for fat metabolism during a fast. Adipose tissue releases fatty acids for oxidation by multiple tissues. The liver oxidizes fatty acids and esterifies remaining fatty acids, exporting them as VLDL. The liver also plays an indispensable role in glucose production; under these conditions, the unique flexibility of the liver works to maintain whole‐body fuel availability even at the expense of its ATP pool (Berglund et al. 2009). This vital response of the liver might, in part, explain the differences in metabolic gene expression between the liver and WAT during fasting (Palou et al. 2008). When transitioning from the fed to the fasted state, the liver rapidly decreases expression of lipogenic genes (*Srebp1c* and *Fasn*) after a short fast (4 h). The lipolytic and fatty acid oxidative genes (i.e. *Ppar‐α*,* Fgf21* and *Cpt‐1*) increase after a longer fast (8 h). Once again, white adipose tissue has a different behavior; a longer fast is required to observe the same alterations in lipogenic (8 h) and lipolytic (24 h) gene expression as is seen in the liver.

The metabolic and molecular physiology of the liver and WAT under fed and fasted conditions has been described in detail (Irimia et al. 2010; Wu et al. 2012). However, the metabolic adaptations of the liver and WAT to differing (but physiologically and clinically relevant) feeding patterns of isocaloric nutritional support, need to be elucidated.

The experiments herein sought to determine the effect of feeding pattern on the metabolic adaptations of liver and WAT. Mice were placed on enteral nutrition for 10 days which was infused at a constant or variable (80% of their daily caloric requirements were given during the night and 20% during the day) rate. Using a novel in vivo approach of chronic isocaloric nutrient delivery, the results demonstrate that the liver's lipogenic response to feeding pattern exceeds that of the WAT in the mouse.

## Methods

### Animals

All procedures were performed on male wild type C57BL/6J mice (Jackson Laboratories, Bar Harbor, ME). At 3 weeks of age, mice were separated by gender and maintained in micro‐isolated cages on a 12/12 h light/dark cycle (06:00/18:00) with free access to food and water. All experiments were performed in mice at ~3 months of age and they were approved by Vanderbilt University Institutional Animal Care and Use Committee.

### Surgical procedures

Mice were anesthetized using isofluorane. Gastric catheters were placed directly via gastrostomy. The jugular vein was also catheterized. Both catheters were tunneled subcutaneously to the back of the neck where the loose ends were attached via stainless steel connectors to tubing made of micro‐renathane (0.033 in OD). The tubing was exteriorized and sealed with stainless steel plugs. Animals were individually housed after surgery. Body weight was recorded daily.

### Experimental design

The day after surgery, mice were placed in Nalgene techniplast rodent metabolic cages to be acclimated and fed with chow (AIN‐76‐Modified, Bio‐Serv). After acclimation period (3 days), animals were randomized to chow plus saline infusion (0.9%, 4 mL/day; *n *= 12) or total enteral nutrition (TEN; *n *= 21). Animals were connected to infusion pumps through a swivel system (Instech) to deliver either saline or TEN enterally. Water was given ad libitum. Mice with enteral nutrition were divided into two groups: continuous enteral nutrition (CN, *n *= 12), where they received the daily caloric intake (15 kcal) at a constant infusion rate for 24 h/day (10 mL/day), or variable enteral nutrition (VN, *n *= 9) where they received 80% of caloric intake during the night and 20% during the day. The groups were subdivided into morning (06:00; Ch6, *n *= 6; CN6, *n *= 6; VN6, *n *= 4) and evening (18:00; Ch18, *n *= 6; CN18, *n *= 6; VN18, *n *= 5) groups according the time they were sacrificed. TEN was composed of glucose, lipids, amino acids (Travasol^®^), saline, and a multivitamin supplement (MVI‐12; Astra USA). Glucose (50% dextrose; Abbott) made up 75% of the nonprotein calories, and a fat emulsion (20% Intralipid^®^; Baxter Healthcare) constituted the remaining 25% of the energy requirements. All the groups received the same daily caloric amount. Chow fed mice were given their entire daily caloric allotment (15 Kcal) at 18:00. Urine was collected daily in a dark recipient, total volume was recorded, 1 mL aliquot was centrifuged to remove sediments and stored at −80°C until analyzed. After 10 days, at either 06:00 or 18:00 mice were anesthetized by IV injection using sodium pentobarbital. Blood samples were taken. Muscle (*soleus, gastrocnemius,* and *superficial vastus lateralis*), liver, white adipose tissue, heart and brain were removed, frozen in liquid nitrogen and stored at −80°C until processing and analysis.

### Quantitative Real Time PCR

Total mRNA was extracted from liver and white adipose tissue using TRIzol^®^ (Invitrogen, Life Technologies). cDNA was synthesized from 2 *μ*g RNA with the High Capacity cDNA transcription kit (Applied Biosystems, Life Technologies). PCR amplification was carried out using the ABI system with Taqman probes and the ΔΔCt method was used to quantify mRNA levels. Gene expression was normalized using *Gapdh* as a housekeeping gene. CLOCK genes and its endogenous control (*Hprt*) were amplified using the SybrGreen method. Table 1 shows all the genes measured. Data are represented using the Rq which is normalized to the morning Chow fed group or Rq = 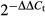 [Δ*C*_t_ = *C*_t_ (target)‐*C*_t_ (*Gapdh*); ΔΔ*C*_t_ = Δ*C*_t_ (sample)‐Δ*Ct* (Ch6)].

**Table 1.** Enzymes and hormones measured

Protein	Abbreviation	Gene
Carbohydrate‐responsive element binding protein	ChREBP	*Mxlpl (*MLX interacting protein*)*
Sterol regulatory element binding protein 1c	SREBP‐1c	*Srebf1* (sterol regulatory element binding transcription factor 1)
Acetyl‐CoA carboxylase‐*α*	ACC‐*α*	*Acca*
Acetyl‐ citrate lyase	ACLY	*Acly*
Fatty acid synthase	FAS	*Fasn*
Peroxisome proliferator activated receptor ‐*α*	PPAR‐*α*	*Ppara*
Peroxisome proliferator activated receptor ‐*γ*	PPAR*γ*	*Pparg*
Fibroblast growth factor 21	FGF21	*Fgf21*
Plasminogen activator inhibitor‐1	PAI‐1	*Serpine*
Inducible nitric oxide synthase	iNOS	*Nos2*
Tumor necrosis factor‐*α*	TNF‐*α*	*Tnfa*
Interleukin10	IL10	*Il10*
Monocyte chemotactic protein ‐1	MCP‐1	*Ccl2 (*Chemokine c‐motif ligand 2)
Cryptochrome 1	CRY1	*Cry1*
Cryptochrome 2	CRY2	*Cry2*
Period homolog 2	PER2	*Per2*

### Western Blot

Protein from liver and adipose tissue was extracted with a buffer containing 50 mmol/L Tris, 1 mmol/L EDTA, 1 mmol/L EGTA, 10% glycerol, 1% Triton X‐100 at pH 7.5. Before using 1 mmol/L DTT, 1 mmol/L PMSF, 5 *μ*g/mL protease inhibitor cocktail, 10 *μ*g/mL trypsin inhibitor, 50 mmol/L NaPP were added to the buffer. Tissues were homogenized on ice and centrifuged for 20 min at 4°C. Protein content was quantified (Bradford 1976) and extracts stored at −80°C until used. Protein extracts (30 *μ*g) were combined with NuPage LDS sample loading buffer and NuPage reducing agent (Invitrogen, Life Technologies), boiled at 95°C for 5 min and size‐fractioned by electrophoresis on SDS‐PAGE gels. Proteins were transferred to PVDF membranes by electroblotting. Membranes were incubated with 5% nonfat dried milk in Tris‐buffered saline‐Tween (TBS‐T) for 1 h, washed 3× for 10 min in TBS‐T and then incubated overnight at 4°C with anti‐GK antibody generously donated by Dr. Masakazu Shiota at Vanderbilt University; anti‐GKRP antibody (sc‐11416 Santa Cruz Biotechnology), anti‐phosphoAMPK (Thr 172) antibody (#2531 Cell Signaling Technology), anti‐AMPK antibody (#2532 Cell Signaling Technology). As a loading control, after the antibody used, every membrane was incubated with anti‐*β*‐actin antibody (#4967 Cell Signaling Technology). After incubation, membranes were washed 3× for 5–10 min in TBS‐T, incubated with peroxidase‐conjugated secondary antibody 1 h at room temperature, and analyzed using enhanced chemiluminescence (GE Healthcare).

### Plasma, urine, and tissue sample analysis

Insulin, glucagon, leptin, and urinary C‐peptide were measured using radioimmunoassay by the Vanderbilt Hormone Assay and Analytical Services Core. Urinary creatinine was assayed using a colorimetric detection kit (Enzo Life Sciences). Lipids were extracted from liver and lipid profile measured by the Hormone Assay and Analytical Services Core. Individual lipid classes were separated by thin layer chromatography and visualized by either iodine vapors or rhodamine 6G, scraped from the plates and eluted from the silica gel. Fatty acids in the triglyceride fraction were quantitated by gas chromatography.

### Histological analysis

Liver was fixed in formalin at room temperature overnight, then dehydrated and embedded in paraffin. Tissue blocks were then sectioned at 5 *μ*m, then stained with hematoxylin and eosin (H&E) following the standard protocols of the Translational Pathology Shared Resource at Vanderbilt University.

### Data analysis

All data were analyzed using Sigma Plot 12.0 (Aspire Software International). Data are presented as the mean ± SEM. Differences between groups were determined by analysis of variance. Paired comparisons were performed with the two‐tailed Student's t‐test. The significance level was set at *P *< 0.05.

## Results

### Whole body metabolic parameters

All cohorts of mice had similar caloric intake and maintained their weight after 10 days of nutrient delivery (Table 2). Plasma glucose and insulin concentrations at 06:00 and 18:00 reflected the feeding pattern (Table 3). In both variable nutrition (VN) and chow (Ch), glucose and insulin levels were higher at 06:00 as compared with 18:00. In contrast, with continuous nutrition (CN), blood glucose and plasma insulin concentrations were unchanged between 06:00 and 18:00. 24‐h urinary C‐peptide excretion (ng/mg creatinine) was threefold greater in Ch (30 ± 7) and VN (32 ± 5) than in CN (11 ± 1; Table 4). After subtracting the estimated insulin secretion during the light phase, nocturnal insulin secretion was increased sixfold in Ch and VN as compared to CN, which reflects a higher rate of nutrient handling during the dark phase in those groups.

**Table 2.** Energy intake and body weight during nutrient delivery (Chow *n *= 12; CN *n *= 12; VN *n *= 9)

Treatment	Initial BW (g)	Final BW (g)	Daily energy intake (kcal/day/g)
Ch	27.8 ± 0.6	28.5 ± 0.8	0.49 ± 0.02
CN	27.6 ± 1	27.1 ± 0.5*	14.7 ± 0.2
VN	28.5 ± 0.9	28.2 ± 0.6	15.5 ± 0.1*

* *P *< 0.05 versus Chow.

**Table 3.** Impact of nutrient delivery on plasma glucose, insulin, glucagon, and leptin concentrations (Ch6 *n *= 6; Ch18 *n *= 6; CN6 *n *= 6; CN18 *n *= 6; VN6 *n *= 4; VN18 *n *= 5)

Group	Glucose (mg/dL)	Insulin (ng/mL)	Glucagon (pg/mL)	Leptin (ng/mL)
Ch6	169 ± 7	3.8 ± 0.9	75 ± 7	0.6 ± 0.1
Ch18	103 ± 9^a^	0.5 ± 0.2^a^	51 ± 7^a^	0.6 ± 0.1
CN6	163 ± 10	4.3 ± 1	59 ± 8^a^	25.9 ± 4^a^^,^^b^
CN18	128 ± 9	4.1 ± 0.7^b^	65 ± 10	18.3 ± 0.8^a^^,^^b^^,^^c^
VN 6	140 ± 15	3.5 ± 1.1	45 ± 1^a^	16.6 ± 0.9^a^^,^^b^^,^^c^
VN18	105 ± 20^a^	0.9 ± 0.3^d^	49 ± 4	8.8 ± 1.2^a^^,^^b^^,^^d^^,^^e^

^a^*P *< 0.05 versus Ch6; ^b^*P *< 0.05 versus Ch18; ^c^*P *< 0.05 versus CN6; ^d^*P *< 0.05 versus CN18; ^e^*P *< 0.05 versus VN6.

**Table 4.** Impact of nutrient delivery on urine volume, C‐peptide, and Creatinine excretion (Ch *n *= 12; CN *n *= 12; VN *n *= 9)

Treatment	24 h urine (mL/day)	24 h C‐peptide (ng/day)	24 h Creatinine (mg/day)	C‐peptide/Creatinine (ng/mg)
Ch	10.6 ± 0.7	23 ± 3.2	1.1 ± 0.3	30.2 ± 7.4
CN	4.7 ± 0.6^a^	8.7 ± 1.3^a^	0.9 ± 0.2	11 ± 1.3^a^
VN	6.3 ± 0.2^a^	20.4 ± 3.6^b^	0.7 ± 0.1	32 ± 5.3^b^

^a^*P *< 0.05 versus Ch; ^b^*P *< 0.05 versus CN.

Glucagon levels were not different among groups. Leptin levels increased in CN and VN compared to Ch.

### Hepatic carbohydrate metabolism

**Figure 1.**
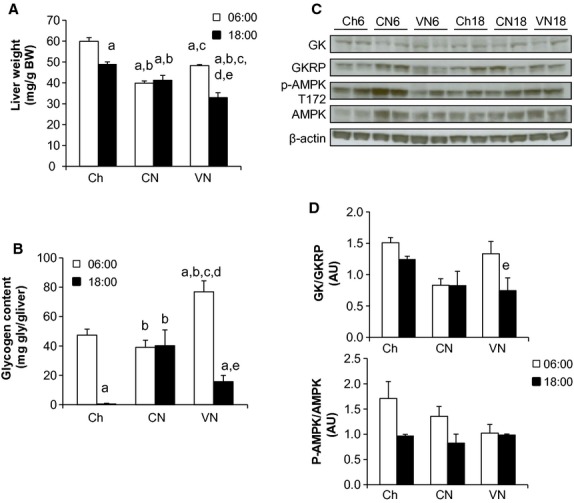
Liver characterization. Liver mass (A), glycogen content (B), glucokinase and glucokinase‐regulatory protein ratio (GK/GKRP) and AMPK phosphorylation state (C and D) at 06:00 and 18:00 in mice fed chow once a day (Ch6 *n* = 6; Ch18 *n* = 6), chronically fed enteral nutrition either continuously either a constant rate (CN6 *n* = 6; CN18 *n* = 6) or a variable rate where 80% of the calories were fed during the dark phase (VN6 *n* = 4; VN18 *n* = 5). Blots are representative experiments from *n* = 3. Data are presented as mean ± SEM ^a^*P *< 0.05 versus Ch6; ^b^*P *< 0.05 versus Ch18; ^c^*P *< 0.05 versus CN6; ^d^*P *< 0.05 versus CN18; ^e^*P *< 0.05 versus VN6.

In all groups during the light hours (06:00 and 18:00), plasma insulin and glucose were similar. In the CN group, liver glycogen content was similar in the morning and evening due to the continuous infusion of enteral nutrition (Fig. 1 A and B). In contrast, both Ch and VN glycogen content decreased by 18:00, as caloric intake during the light period was less than energy requirements. Not surprisingly, in CN glucokinase (GK) and glucokinase‐regulatory protein (GKRP; Fig. 1C), glycogen synthase (GS), glycogen synthase kinase‐3*β* (GSK‐3*β*; data not shown) were not altered between 06:00 and 18:00. In contrast, Ch and VN had a different profile. GK/GKRP ratio decreased at 18:00 in both groups, which paralleled the decrease in hepatic glycogen content (Fig. 1D).

### Hepatic lipid metabolism

**Figure 2.**
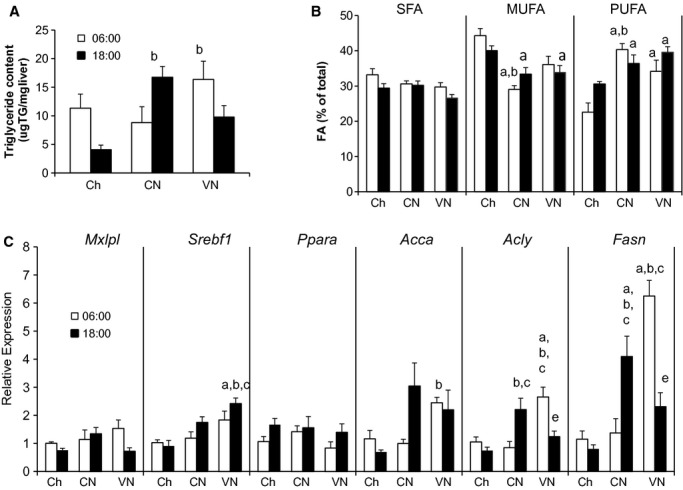
Hepatic lipid metabolism. Triglyceride (A), fatty acid (B) content, and lipogenesis profile gene expression (C) at 06:00 and 18:00 in mice fed chow once a day (Ch), chronically fed enteral nutrition either continuously either a constant rate (CN) or a variable rate where 80% of the calories were fed during the dark phase (VN). *Mlxipl* (ChREBP), *Srebf1* (SREBP‐1c), *Ppara* (PPAR‐*α*), *Acca* (Acetil‐CoA carboxilase)*, Acly* (ATP cytrate lyase), and *Fasn* (Fatty acid synthase). Data represented as mean ± SEM of relative expression (Rq) related to Ch6. Ch6 *n* = 6; Ch18 *n* = 6; CN6 *n* = 6; CN18 *n* = 6; VN6 *n* = 4; VN18 *n* = 5. ^a^*P *< 0.05 versus Ch6; ^b^*P *< 0.05 versus Ch18; ^c^*P *< 0.05 versus CN6; ^d^*P *< 0.05 versus CN18; ^e^*P *< 0.05 versus VN6.

Despite the constant infusion of nutrients in CN, hepatic triglyceride (TG) content (Fig. 2A) was highest at 18:00. In contrast, in both Ch and VN, hepatic TG was highest at 06:00. The change in TG content was reflected in all three categories of fatty acids (SFA, MUFA, and PUFA; Fig. 2B). The individual fatty acids that comprise those fractions are included in Table 5. In general, individual FFA content paralleled the changes in total TG content.

**Table 5.** Fatty acid hepatic composition

FA (*μ*g/mg liver)	Ch6	Ch18	CN6	CN18	VN6	VN18
14:0	10 ± 2	2 ± 1^a^	7 ± 3	15 ± 5^b^	10 ± 3	6 ± 1^b^
16:0 (Palmitate)	342 ± 84	104 ± 20^a^	249 ± 84	455 ± 56^b^	450 ± 94	231 ± 55^b^^,^^d^
16:1 (palmitoleic)	51 ± 16	14 ± 4^a^	30 ± 13	80 ± 35	56 ± 22	27 ± 6
18:0 (stearic)	32 ± 6	13 ± 1^a^	22 ± 6	38 ± 6^b^	32 ± 7	27 ± 5^b^
18:1 (oleate)	470 ± 121	154 ± 33^a^	231 ± 72	489 ± 65^b^^,^^c^	526 ± 86^c^	290 ± 44^b^^,^^d^^,^^e^
18:2 (linoleic)	191 ± 28	96 ± 17^a^	273 ± 85	480 ± 45^b^	449 ± 109*	320 ± 76^b^
18:3 (Linolenic)	9 ± 2	2 ± 1^a^	29 ± 11	58 ± 5^b^^,^^c^	51 ± 13^a^	32 ± 9^b^^,^^d^
20:3w6	8 ± 2	5 ± 1	5 ± 2	10 ± 1^b^	12 ± 4	7 ± 1
20:4 (Arachidonic)	13 ± 1	13 ± 1	13 ± 2	18 ± 2	21 ± 5	12 ± 2
20:5	0	1 ± 1	5 ± 2^a^	7 ± 1^b^	8 ± 2^a^	3 ± 2^b^^,^^e^
22:5w3	1 ± 0	1 ± 1	6 ± 2^a^	10 ± 1^b^	10 ± 3^a^	6 ± 2^b^
22:6	6 ± 1	6 ± 1	10 ± 2	16 ± 2^b^	14 ± 5	17 ± 4^b^

^a^*P *< 0.05 versus Ch6; ^b^*P *< 0.05 versus Ch18; ^c^*P *< 0.05 versus CN6; ^d^*P *< 0.05 versus CN18; ^e^*P *< 0.05 versus VN6.

While PPAR‐*α* gene expression (Fig. 2C) was not significantly altered by diet or time of day, the expression of lipogenic genes were markedly dependent on the pattern of nutrient delivery. Both CN and VN cohorts had an augmented hepatic lipogenic capacity; however, the timing of the increase was dependent on the pattern of nutrient delivery. In parallel with the higher TG content, lipogenic gene expression of acetate citrate lyase (*Acly*), acetyl‐CoA carboxylase‐*α* (*Acca*) and fatty acid synthetase (*Fasn*) were highest at 18:00 in CN. Moreover, the phosphorylation state AMPK (Fig. 1C and D) was decreased, which could also contribute to sustained lipogenesis.

On the other hand, in VN, hepatic TG content was lowest at 18:00. This was paralleled by decreases in *Fasn* and *Acly* expression. *Acca*, while increased above the Ch, did not decrease at 18:00. Interestingly, decreases in *Fasn* and *Acly* could not be explained by a fall in sterol regulatory element‐binding protein 1c (SREBP‐1c) gene expression (*Srebf1*); carbohydrate response element binding protein (ChREBP) gene expression (*Mlxipl*) also did not significantly decrease. The phosphorylation status of AMPK was not markedly different between 06:00 and 18:00 in VN. Despite the increase in lipogenesis, there was no histological indication of steatosis or fat accumulation (data not shown).

**Figure 3.**
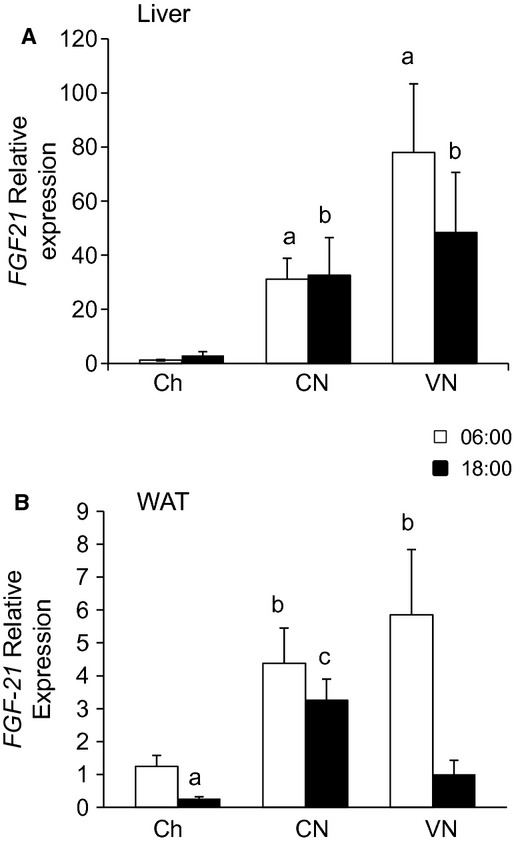
FGF21 gene expression. Liver (a) and white adipose tissue (b) FGF21 gene expression in mice fed chow one a day (Ch), chronically fed enteral nutrition in a constant (CN) or variable (VN) rate at 06:00 or 18:00. Data represented as mean ± SEM of relative expression (Rq) related to Ch6. Ch6 *n* = 6; Ch18 *n* = 6; CN6 *n* = 6; CN18 *n* = 6; VN6 *n* = 4; VN18 *n *= 5. ^a^*P *< 0.05 versus Ch6; ^b^*P *< 0.05 versus Ch18; ^c^*P *< 0.05 versus CN6; ^d^*P *< 0.05 versus CN18; ^e^*P *< 0.05 versus VN6.

Nutrient delivery dramatically increased FGF21 expression in the liver in both CN and VN (Fig. 3A). While lipogenic capacity of the liver was very sensitive to the pattern of nutrient delivery, FGF21 expression was not.

### WAT metabolism

**Figure 4.**
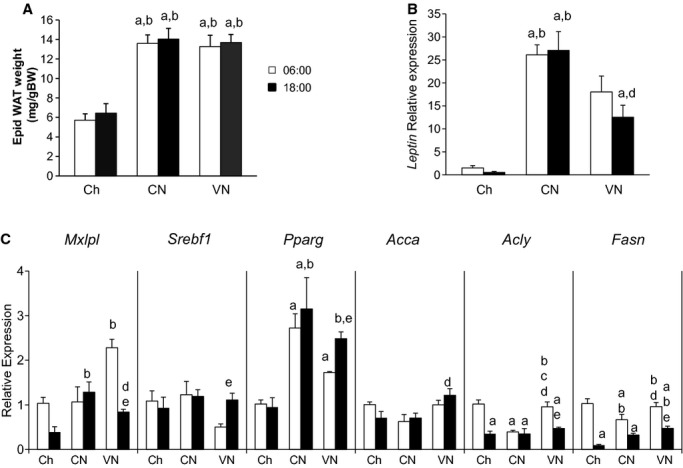
White adipose tissue characterization. WAT mass (A), leptin (B) and lipogenesis (C) gene expression in mice fed chow one a day (Ch), chronically fed enteral nutrition in a constant (CN) or variable (VN) rate at 06:00 or 18:00. *Mlxipl* (ChREBP), *Srebf1* (SREBP‐1c), *Pparg* (PPAR‐*γ*), *Acca* (Acetil‐CoA carboxilase)*, Acly* (ATP cytrate lyase) and *Fasn* (Fatty acid synthase).Data represented as mean ± SEM of relative expression (Rq) related to Ch6. Ch6 *n *= 6; Ch18 *n *= 6; CN6 *n *= 6; CN18 *n *= 6; VN6 *n *= 4; VN18 *n *= 5. ^a^*P *< 0.05 versus Ch6; ^b^*P *< 0.05 versus Ch18; ^c^*P *< 0.05 versus CN6; ^d^*P *< 0.05 versus CN18; ^e^*P *< 0.05 versus VN6.

Consistent with the increase in plasma leptin, epididymal adipose tissue content (Fig. 4A) and leptin gene expression (Fig. 4B) increased in both CN and VN relative to Ch; the extent of the adiposity was not altered by the pattern of nutrient delivery. As was observed in the liver, adipose tissue FGF21 expression was increased in all the groups with TEN, although to a lesser extent (Fig. 3B). The expression was highest in the morning and decreased at 18:00. In a separate cohort of mice, adiposity did not increase in animals given the nutrition only once a day (11 ± 2 vs. 9 ± 1 mg/g BW; Chow vs. TEN). Despite the increase in adipose tissue mass, lipogenic capacity in WAT did not markedly increase. In addition, unlike the liver, the adipose tissue lipogenic activity did not follow the pattern of nutrient delivery. *Acly* and *Fasn* were highest in the morning in all groups except in CN. *Acca* expression was not altered. Surprisingly, the gene expression of SREBP‐1c (*Srebf1*) and PPAR‐*γ* (*Pparg*) could not explain the pattern of lipogenic gene expression. SREBP‐1c and PPAR‐*γ* expression increased at the end of the light phase; this increase was more marked in the VN group. Once again, lipogenic gene expression paralleled the expression of its regulator, ChREBP (*Mlxipl*).

**Figure 5.**
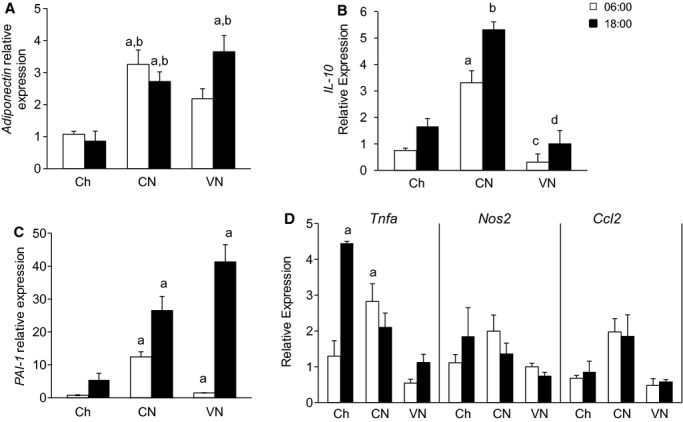
White adipose tissue inflammation. Anti‐ (A and B), and pro‐ (C and D) inflammatory gene expression in the WAT of mice fed chow one a day (Ch), chronically fed enteral nutrition in a constant (CN) or variable (VN) rate at 06:00 or 18:00. *IL‐10* (interleukin‐10), *PAI‐1* (plasminogen activator inhibitor‐1), *Tnfa* (Tumor necrosis factor‐*α*), *Nos2* (inducible nitric oxidase), *Ccl2* (chemokine ligand‐2), *Il‐10* (interleukin‐10)).Data represented as mean ± SEM of relative expression (Rq) related to Ch6. Ch6 *n *= 6; Ch18 *n *= 6; CN6 *n *= 6; CN18 *n *= 6; VN6 *n *= 4; VN18 *n *= 5. ^a^*P *< 0.05 versus Ch6; ^b^*P *< 0.05 versus Ch18; ^c^*P *< 0.05 versus CN6; ^d^*P *< 0.05 versus CN18; ^e^*P *< 0.05 versus VN6.

In parallel with the increase in leptin expression and WAT mass, adiponectin and PAI‐1 gene expression were also markedly increased, with the highest expression at 18:00 (Fig. 5A and C). Other pro‐ and anti‐inflammatory markers (*Tnfa, Nos2, Ccl2,* and *Il‐10* respectively) did not show significant changes in expression (Fig. 5B and D).

**Figure 6.**
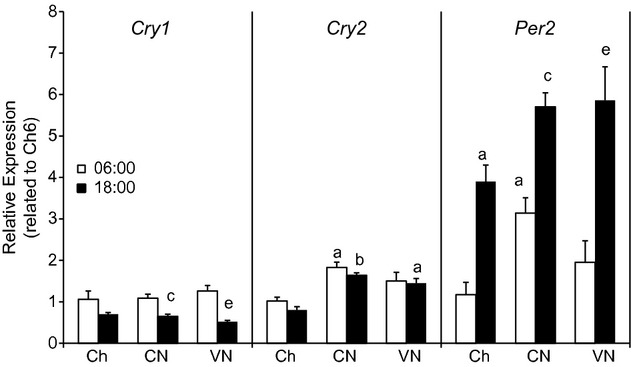
Circadian rhythm in WAT. CLOCK (*Cry1, Cry2, and Per2*) gene expression in the WAT of mice fed chow once a day (Ch), chronically fed enteral nutrition in a constant (CN) or variable (VN) rate at 06:00 or 18:00. *Cry1* (Cryptochrome 1), *Cry2* (Cryptochrome 2), *Per2* (Period circadian protein homolog 2). Data represented as mean ± SEM of relative expression (Rq) related to Ch6. Ch6 *n *= 6; Ch18 *n *= 6; CN6 *n *= 6; CN18 *n *= 6; VN6 *n *= 4; VN18 *n *= 5. ^a^*P *< 0.05 versus Ch6; ^b^*P *< 0.05 versus Ch18; ^c^*P *< 0.05 versus CN6; ^d^*P *< 0.05 versus CN18; ^e^*P *< 0.05 versus VN6.

The expression of CLOCK genes (Fig. 6), which control the circadian rhythm, showed the expected changes between morning and night (decreased *Cry1* and increased *Per2*). *Cry2* was significantly increased in both groups with constant infusion (CN6, CN18) compared with Chow groups but there were not differences among the other groups.

## Discussion

In this study, we assessed hepatic and white adipose tissue adaptations to long‐term enteral nutrition. Nutrition was given either continuously or in a pattern that reflects the nocturnal feeding behavior of mice. We observed that by switching animals to an elemental diet (TEN) with a comparable macronutrient profile and caloric content to chow fed animals, total adipose tissue mass as well as the expression of adiponectin and leptin increased irrespective of the feeding pattern (CN or VN). Despite increases in adiposity with enteral nutrition, inflammation and whole body insulin requirements were unaltered in either continuous or variable nutrition‐treated animals. While hepatic carbohydrate, lipid accumulation and daily insulin secretion rapidly responded to the pattern of nutrient delivery metabolism, the regulation of adipose tissue lipid metabolism remained insensitive. Thus, the uninterrupted enteral delivery of isocaloric nutrition predisposed mice to lipid accumulation in white adipose tissue; this, however, may not necessarily have led to insulin resistance. While the extent to which the adiposity increased was not sensitive to the pattern of nutrient delivery, the overall insulin requirements were substantially lower when nutrients were infused in a continuous rather than a discontinuous pattern.

Switching animals to an isocaloric, elemental diet (total enteral nutrition, TEN) predisposes the animals to lipid accumulation in adipose tissue. We observed that 10 days of TEN increased epididymal fat pad mass (1.8–fold). This was associated with a 20‐fold increase in both plasma leptin concentration and adipose tissue leptin expression, which could be explained by the lack of interruption of the dietary intervention. The mechanism for this is unclear as the total caloric content and composition of the diets were very similar. It is possible that whole body energy expenditure was actually lower in the animals receiving TEN; however, giving the animals the elemental diet once a day did not cause lipid accumulation. Also, the bioavailability of the calories may differ. With the chow diet, the energy required to digest the nutrients in the chow may have offset any net caloric effect of the diet. While the diets were not identical, the fatty acid composition (corn oil vs. soybean oil) and carbohydrate (maltodextrose vs. glucose) content were very similar. An additional hypothesis is that as both TEN groups never went into a complete fasting state. The pattern of fuel partitioning was altered such that a greater portion of the dietary nutrients were deposited as lipid during the first few days of nutrition as they adapted to the differing nutrients. Also, the alteration of feeding pattern could have increased adiposity by inhibiting the phosphate pentose pathway, as it has been previously described (Stucchi et al. 2012). However, this observation was made with a high fat diet.

Despite the increase in adiposity, animals receiving TEN (either CN or VN) were not insulin resistant. 24‐h insulin secretion (i.e. C‐peptide excretion) in the VN and chow‐fed groups were identical. This suggests that while TEN predisposed the animals to gain adipose tissue, mice did not develop insulin resistance. A number of factors may have limited the development of insulin resistance. The dietary fat represented only 25% of the nonprotein calories in TEN. The content of saturated fatty acids in intralipid is small (soybean oil; <15% of total lipid) and the oil is rich in mono and poly unsaturated fats. While acute intravenous infusion of intralipid combined with heparin has been shown to induce insulin resistance by increasing circulating nonesterified fatty acids (Charbonneau and Marette 2010), an increase in nonesterified fatty acids would not be expected when lipid is given enterally as they will be packaged in chylomicrons. Another possibility as to why insulin resistance was not observed is that mice fed with TEN had a significant increase of hepatic omega‐3 PUFA (i.e. eicosapentanoic acid, Table 5) which are anti‐inflammatory and can improve insulin action (Kalupahana et al. 2011; Gillies et al. 2012; Rius et al. 2012).

Surprisingly, the slight increase in gene expression of pro‐inflammatory cytokines (*Tnfa*,* Nos2*,* Ccl2*) in the TEN groups was accompanied by increases in anti‐inflammatory markers (*IL‐10* and *Adiponectin*; Fig. 5). This suggests that the TEN groups developed a healthy adiposity. Most models of obesity exhibit an increase in leptin and a decrease in adiponectin (Enos et al. 2013). However, our model is similar to that seen with PPAR*γ* agonists where both adiponectin and leptin expression increase (Hammarstedt et al. 2005). Moreover, we observed a marked increase in hepatic and adipose expression of FGF21, which may have augmented PPAR*γ* associated activity and explained the accompanying increase in adiponectin (Cuevas‐Ramos et al. 2012; Liu and Liu 2012). The increase in adipose tissue FGF21 gene expression is likely driven by the persistent glucose availability during the light hours in both the constant and variable TEN infusion (Sanchez et al. 2009).

Delivering a constant infusion of nutrients markedly decreased whole body insulin requirements. Animals receiving a constant infusion of TEN required only one‐third of the total insulin requirement of both the chow‐fed and VN groups. Since circulating insulin concentrations between 06:00 and 18:00 were relatively stable, we predicted the average insulin concentrations during the dark phase from the urinary C‐peptide excretion. In the constant infusion group, average arterial insulin concentration remained at 3 ng/mL throughout the day. In contrast, arterial insulin concentration during the dark phase in VN and Ch groups, was predicted to be over 20 ng/mL. These results are similar to observations we made in dogs receiving chronic nutritional support (Chen et al. 2005a,b). Constant infusion of nutrients promotes caloric oxidation with very little storage as the calories infused meet the immediate metabolic demands. In contrast, during the dark phase in VN and Ch groups, caloric intake exceeds caloric demands, resulting in activation of glycogen synthesis and lipid synthesis. This requires additional activation of insulin secretion. The average energy needs of the mouse demanded carbohydrate infusion at an average rate of 90 mg/kg/min in the constant infusion group, resulting in prevailing insulin concentration of 3 ng/mL. In the Ch and VN groups, glucose absorption was over 145 mg/kg/min during the dark phase. As maximal insulin‐stimulated glucose utilization at euglycemia in the mouse is approximately 100 mg/kg/min, the increase in glucose levels will synergize with the accompanying increase in insulin to augment glucose utilization and storage in muscle, liver, and adipose tissue. This supports the use of low glycemic index enteral nutrition for hospitalized patients, which would essentially smooth out carbohydrate absorption and decrease insulin requirements and lower risk of hyperglycemia, especially in sensitive populations (Ceriello et al. 2009; Vaquerizo Alonso et al. 2011).

Interestingly, hepatic carbohydrate metabolism was very sensitive to the pattern of nutrient delivery. As expected, hepatic glycogen content was stable in animals on continuous nutrition (CN), while in the chow and variable TEN (VN) groups, glycogen content was highest at 06:00 and lowest at 18:00. Remarkably, in the VN group, the hepatic glycogen content decreased between 06:00 and 18:00, despite the fact that the glucose infusion rate during the light phase was 36 mg/kg/min. This is twice the basal glucose disposal of a 5–6 h fasted mouse. Continuous infusion of nutrients given enterally or parenterally has been shown to augment hepatic glucose utilization, which occurs within 24 h of the onset of nutrition (Chen et al. 2000, 2005b). In this context, we did not observe an increase in GK or decreases in GS or GSK‐3 phosphorylation. However, we did observe a decrease in GK in both VN and chow groups as the animals transitioned to a fast. We suspect from the absolute decrease in hepatic glycogen content between 06:00 and 18:00 that hepatic glycogenolysis was at a value of at least 3 mg/kg/min. As whole body glucose utilization in the post absorptive state is approximately 15–20 mg/kg/min; it is likely that gluconeogenesis was also contributing to hepatic glucose production.

The impact of the pattern of nutrient delivery on hepatic and adipose lipid metabolism was dramatically different. The liver was highly responsive to the pattern of nutrient delivery. In the continuous infusion group, hepatic lipid content was highest at 18:00, while in VN it was highest at 06:00. This paralleled the marked changes in gene expression of *Fasn* and other lipogenic enzymes. The data for the continuous infusion group suggest fuel partitioning to TG in the liver is entrained to occur in the afternoon (Jang et al. 2012). Indices of hepatic lipid metabolism (i.e. AMPK) were lower which also could contribute to the increase in hepatic TG. Delivering the majority of the calories in the evening in VN led to an increase in insulin combined with increased lipogenic capacity and carbohydrate availability. This shifts the response to the nocturnal feeding period, explaining the higher TG hepatic content in VN at 6:00. This is associated with accompanying increases in the gene expression of SREBP‐1c (*Srebf1*), *Fasn*, and *Acly*. In contrast, in adipose tissue, lipid synthetic capacity (*Fasn, Acly*) was lowest in the evening and was not modulated by the feeding pattern. A recent study suggests that dietary fat can modulate the expression of peripheral circadian CLOCK genes (Jang et al. 2012). However, we found the expression of CLOCK genes (*Cry1*,* Cry2,* and *Per2*; Fig. 6) in the adipose tissue was not responsive to the TEN feeding pattern, despite increases in the expression of *Cry2* and *Per2*.

SREBP‐1c, a modulator of insulin‐induced lipogenic gene expression, could not explain the accompanying changes. On the other hand, the carbohydrate‐responsive regulator of lipogenic genes (ChREBP) exhibited small changes but in a similar pattern as lipogenic genes. Thus, either SREBP‐1c or ChREBP appear to control lipogenesis in our model, suggesting that factors such as changes in autonomic drive may be important in patterning lipogenic gene expression in adipose tissue.

FGF21 may provide insight into the mechanisms driving our results. FGF21 is traditionally described as a fasting hormone (Potthoff et al. 2009), but there is evidence that FGF21 also increases in the fed state (Uebanso et al. 2011). The regulation of FGF21 during fasting is contradictory; PGC‐1*α* negatively regulates FGF21 (Estall et al. 2009), while PPAR*α* augments the expression of FGF21 (Badman et al. 2007), and both PGC‐1*α* and PPAR*α* increase during fasting. Mice fed TEN had elevated PPAR*α* gene expression (both in liver and WAT) that paralleled the changes in FGF21 gene expression. Thus, PPAR*α* could be regulating the expression of this growth factor. Also, hepatic PGC‐1*α* expression should be decreased during the fed state, so that could contribute to the large increase in hepatic FGF21 gene expression. Recent data suggest that FGF21 increases adiponectin release by adipose tissue (Holland et al. 2013; Lin et al. 2013), which may play an important role in sustaining glucose disposal and minimizing insulin requirements. This relationship between FGF21 and adiponectin would also explain the increase of this adipokine observed in our model despite the increase in leptin.

## Conclusions

In summary, liver and white adipose tissue respond differently to the pattern of nutrient delivery. While hepatic metabolism adapts to the pattern of nutrient availability, white adipose tissue does not. The ability of the white adipose tissue to adapt to a change in diet but not to the pattern of nutrient delivery is advantageous, as the major role of adipose tissue is to store excess calories as lipid to prevent deposition in other tissues (liver and muscle), which could negatively impact their function. Moreover, sustained nutrient delivery even when isocaloric, can increase adiposity, although the proinflammatory profile observed in hypercaloric feeding is not observed. Interestingly, sustained nutrient availability can lower overall insulin requirements independent of changes in FGF21 or adiponectin availability. This work will help to understand the effect that alterations in the pattern of nutrition delivery has on whole body metabolism, especially in the clinical setting where nutritional support is commonly used.

## Acknowledgments

GK antibody and CLOCK genes primers were kindly donated by Dr. Masakazu Shiota and Dr. Carl Johnson from Vanderbilt University respectively.

## Conflict of interests

None declared.
